# Enhanced predictive accuracy of mortality in VLBW infants with late-onset sepsis through a time-specific nomogram

**DOI:** 10.3389/fpubh.2025.1548695

**Published:** 2025-04-02

**Authors:** Lun Yu, Yanhong Li, Yang Zuming

**Affiliations:** ^1^Suzhou Municipal Hospital, Suzhou, China; ^2^Children's Hospital of Soochow University, Suzhou, Jiangsu Province, China

**Keywords:** neonatal late-onset sepsis, nomograms, very-low-birth-weight (VLBW) infants, prediction model, COVID-19

## Abstract

**Objective:**

This study aims to develop and validate a nomogram-based scoring system to predict mortality in very low birth weight (VLBW) infants with late-onset sepsis (LOS). Timely risk stratification in this vulnerable population is critical for optimizing clinical outcomes.

**Methods:**

We conducted a retrospective analysis on 202 VLBW infants diagnosed with LOS between January 2018 and December 2022. Predictive models were created at three key time points: 0 h, 6 h, and 12 h post-sepsis onset, utilizing Least Absolute Shrinkage and Selection Operator (LASSO) regression for variable selection and multivariable logistic regression for model construction. Internal validation was performed with 1,000 bootstrap resamples to correct for potential overfitting. External validation was conducted on an independent cohort of 71 infants from January 2023 to March 2024. Model performance was assessed using Area Under the Curve (AUC), calibration plots, and decision curve analysis (DCA).

**Results:**

The models exhibited excellent discrimination with AUCs of 0.83, 0.92, and 0.94 at 0 h, 6 h, and 12 h, respectively, in the development cohort, and 0.95, 0.95, and 0.97 in the validation cohort. Calibration plots showed strong agreement between predicted and observed outcomes. The significant disparity in maternal COVID-19 infection rates between cohorts (1 vs. 89%) may have contributed to the enhanced predictive accuracy in the external cohort.

**Conclusion:**

This dynamic, time-specific nomogram demonstrates high predictive accuracy and clinical utility for mortality in VLBW infants with LOS. The impact of maternal COVID-19 infection on neonatal outcomes offers a novel perspective for future research in sepsis prognostication.

## 1 Introductions

Late-onset sepsis (LOS) represents a critical infectious condition associated with significant morbidity and mortality rates within neonatal intensive care units (NICUs), particularly affecting preterm infants, especially those with very low birth weight (VLBW) (1). These infants exhibit heightened susceptibility due to their underdeveloped immune systems and extended hospitalizations, which increase their exposure to nosocomial pathogens (2). LOS in VLBW infants is associated with numerous severe complications, including bronchopulmonary dysplasia, intraventricular hemorrhage, necrotizing enterocolitis, and neurodevelopmental impairments (3). Despite advancements in neonatal care, the mortality rate associated with LOS remains considerable, ranging from 5 to 20% (4), necessitating the development of more accurate prediction tools to aid clinical decision-making. Furthermore, survivors are at an elevated risk for long-term disabilities, thereby exacerbating both clinical and financial burdens (5).

Diagnosing and predicting late-onset sepsis (LOS) in neonates presents significant challenges due to the non-specific nature of its symptoms, which frequently overlap with those of other neonatal conditions, resulting in treatment delays. Current diagnostic modalities, such as blood cultures, are limited by prolonged turnaround times and suboptimal sensitivity, highlighting the necessity for more rapid and reliable prediction approaches (6). Various predictive models have been developed that incorporate clinical signs, biomarkers (e.g., C-reactive protein, procalcitonin), and demographic data to assess the risk of sepsis and associated mortality (7, 8). However, these models often lack real-time updates and do not adequately reflect the dynamic progression of sepsis. As a result, they may overlook critical, time-sensitive changes in a patient's condition that are vital for informing timely therapeutic interventions. These limitations emphasize the pressing need for a more advanced tool that not only integrates static clinical parameters but also continuously monitors the evolving disease process, thereby facilitating prompt interventions and improving prognostic accuracy.

This study aims to address these gaps by developing and validating a nomogram-based scoring system specifically designed to predict mortality in VLBW infants with LOS. This model is unique in that it leverages time-dependent variables at three critical time points (0 h, 6 h, and 12 h post-onset of sepsis), allowing for continuous risk stratification as the infant's condition evolves. Such an approach enables clinicians to anticipate clinical deterioration more accurately and adapt treatment protocols accordingly. Furthermore, this study seeks to validate the model both in internally and externally, using an independent cohort with a markedly different maternal COVID-19 exposure rate, thereby testing the model's robustness across different clinical contexts.

The innovation of this study lies in two key aspects: first, the time-sensitive nature of the model, which allows for dynamic risk prediction and clinical management adjustments during the critical early phases of LOS; and second, the exploration of the potential influence of maternal COVID-19 infection on neonatal outcomes in VLBW infants. The stark difference in COVID-19 exposure between the development and external validation cohorts provides a unique opportunity to investigate how maternal viral infections might impact neonatal susceptibility and outcomes in the context of sepsis. To our knowledge, this is one of the first studies to integrate these two novel components into a sepsis mortality prediction model for VLBW infants, providing both immediate clinical relevance and contributing new insights to the broader field of neonatal sepsis research.

## 2 Methods

### 2.1 Study cohort

This study was designed as a retrospective analysis of data collected from neonates diagnosed with late-onset sepsis who were admitted to Suzhou Municipal Hospital. The dataset was divided into a training set and a test set based on the chronological order of data collection. Specifically, neonates admitted between January 1, 2018, and December 31, 2022, while those admitted between January 1, 2023, and April 30, 2024 formed the test set. This temporal split was employed to mimic a real-world prospective scenario, where the model, developed using past data, is validated on more recent, unseen data.

**Figure 1:**
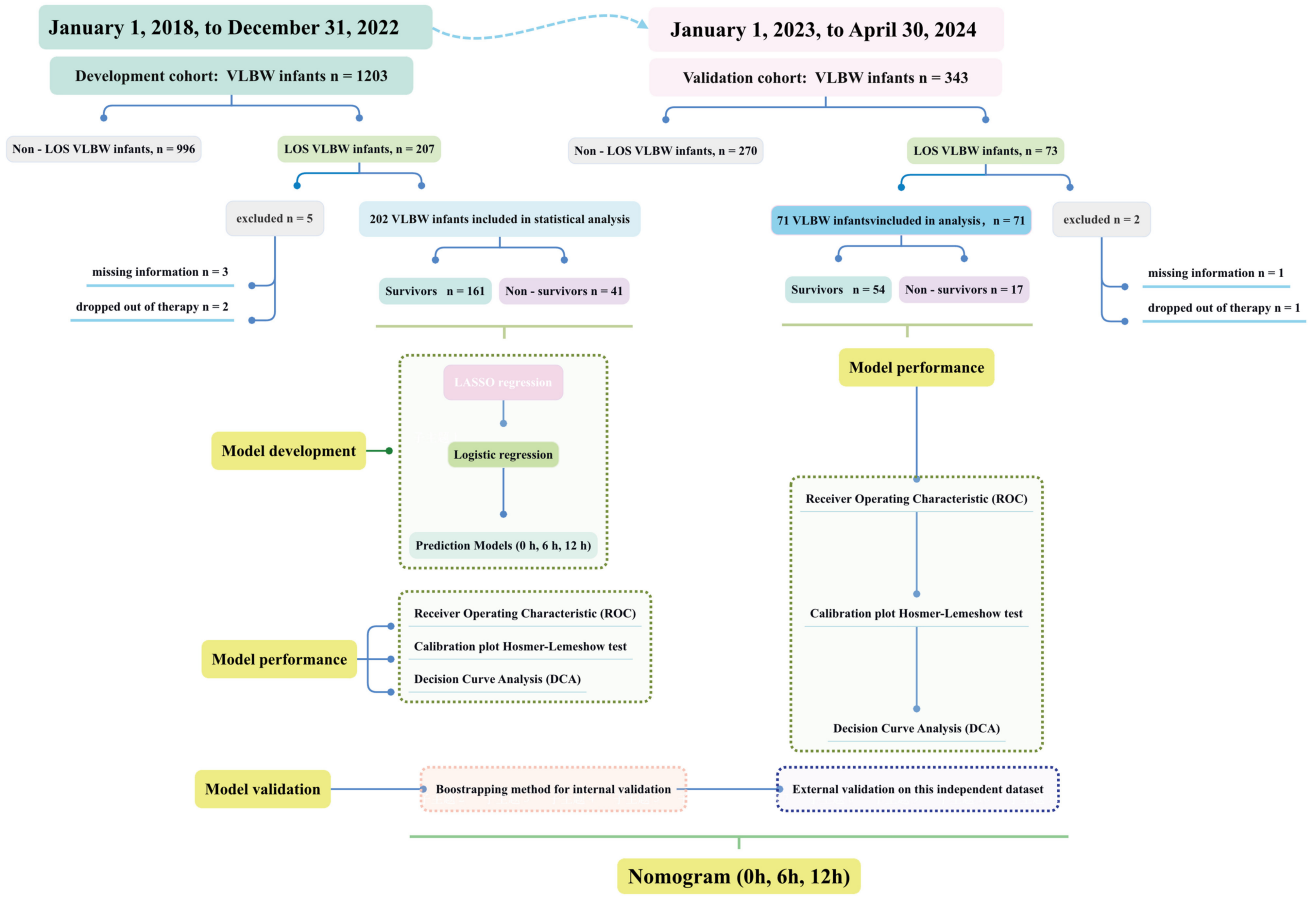
Flow chart for the participants and research process.

Inclusion criteria were as follows: (a) gestational age of ≤ 32 weeks and (b) birth weight < 1,500 g. Exclusion criteria included: (a) infants with missing information and (b) infants whose guardians abandoned or withdrew them from treatment within 7 days of the onset of sepsis. A flowchart was provided to illustrate the detailed process (Figure 1). The analysis was conducted in accordance with the TRIPOD statement (9).

### 2.2 Data collection

Clinical characteristics and laboratory results were obtained from the hospital's electronic medical record system, following a predetermined protocol. The data collection encompassed the following categories: (1) general data, including gender, gestational age, birth weight, delivery method, and Apgar score; (2) clinical manifestations, such as apneic episodes, lethargy, tachycardia or bradycardia, abdominal distension, and skin perfusion abnormalities indicative of microcirculatory irregularities; (3) laboratory results, comprising routine blood tests (white blood cell counts, absolute neutrophil counts, and platelet counts), biochemical indices (procalcitonin and C-reactive protein), and arterial blood gas analyses (pH values, blood lactate concentration, sodium concentration, and blood glucose levels), along with blood culture results; (4) complications associated with sepsis and other prognostic factors (4), including necrotizing enterocolitis (≥ stage II) (10), pulmonary hemorrhage (11), septic shock (12), the neonatal Sequential Organ Failure Assessment (nSOFA) score (13), modes of respiratory support (14), and the oxygenation index (OI), calculated as [(FiO_2_ × Mean airway pressure × 100) ÷ PaO_2_]. Further details and additional information are provided in Supplementary Table S1.

### 2.3 Definitions

LOS was defined as (1) sepsis occurring after 72 h of life (1), (2) blood culture was drawn, and empirical antimicrobial therapy was initiated at the time of evaluation, continuing for a minimum of 5 days or until the patient's demise (13). Sepsis episodes were defined by the occurrence of acute clinical deteriorations indicative of suspected infection, with the 0-h time point established based on specific clinical manifestations, including: (1) hypothermia or hyperthermia; (2) hypotonia or lethargy; (3) tachycardia or bradycardia; (4) increased frequency of apnea or the emergence of new episodes; (5) an elevated requirement for respiratory support; (6) abdominal distension accompanied by decreased feeding tolerance; (7) poor peripheral perfusion, characterized by pale, cold, and mottled skin; and (8) glucose instability, presenting as either hyperglycemia or hypoglycemia (5). At this 0-h time point, prior to the initiation of antibiotic therapy, a minimum volume of 1 mL of peripheral blood was collected from the infant using aseptic techniques to facilitate the isolation of bacterial or fungal pathogens from the bloodstream (4, 13). Subsequent time points were designated at 6 and 12 h following this initial reference.

The earliest manifestation of necrotizing enterocolitis (NEC) and pulmonary hemorrhage in the training set occurred after the diagnosis of suspected sepsis (i.e., after the 0-h time point). Consequently, at the 0-h mark, neither NEC nor pulmonary hemorrhage had yet presented, ensuring that these complications were not present during the initial feature selection phase via LASSO regression. If these complications emerged within 6 h post-onset, they were incorporated as covariates into the 6-h time-point model. Additionally, should they occur between 6 to 12 h post-onset, they were included in the 12-h time-point model to reflect their temporal relationship with the onset of sepsis. This structured approach ensured that the temporal dynamics of complications were appropriately captured and integrated into the respective predictive models.

Components of the nSOFA score include (1) mechanical ventilation and oxygen requirement (score range 0–8), (2) systemic inotropic and steroid application (score range 0 to 4), (3) severity of thrombocytopenia (score range 0 to 3). Based on an online calculator, each infant's nSOFA scores at different time points were calculated (http://www.peds.ufl.edu/apps/nsofa/default.aspx) (13).

### 2.4 Outcome measurements

Sepsis-related deaths were defined as any fatalities occurring within 7 days of a positive blood culture or in the presence of clinical and laboratory evidence of sepsis despite negative blood cultures (8, 15). The primary objective of this study was to determine the incidence of sepsis-related mortality among VLBW infants.

### 2.5 Feature selection and modeling

A two-step process was required to select the component for the prediction model. Firstly, the least absolute shrinkage and selection operator (LASSO) regression algorithm was applied to screen for features related to prognosis for minimizing overfitting. “Lambda.1se” selects the most regularized model within one standard error of the lambda that minimizes cross-validation error. This approach enhances model sparsity and generalizability, reducing overfitting while retaining key predictors. In 10-fold cross-validation, we confirmed that “Lambda.1se” was the optimal tuning parameter (λ), with the least number of variables and the greatest lambda, contributing to achieving the highest prediction performance (16). The variables retained after LASSO regularization, indicated by non-zero coefficients, were included in the final logistic regression model. The variables retained after LASSO regularization, indicated by non-zero coefficients, were included in the final logistic regression model.

Following variable selection by LASSO, a multivariable logistic regression was conducted to further refine the prediction model and estimate the adjusted odds ratios (ORs) for sepsis-related mortality. Both forward and backward selection strategies were used, with a significance level of 0.05 for variable entry and retention in the model. The stepwise approach allowed for the identification of the most statistically significant predictors, while also adjusting for potential confounders.

### 2.6 Model performance and validation

The model's performance was evaluated in terms of discrimination, calibration, and clinical utility. Discriminative ability was measured using the area under the ROC curve (AUC-ROC), with values between 0.7 and 0.9 indicating acceptable to excellent discrimination between survivors and non-survivors. Calibration was assessed using the Hosmer-Lemeshow test (*p* > 0.05 indicating a good fit) and further confirmed with calibration plots to visually compare predicted and observed outcomes.

Internal validation was conducted using 1,000 bootstrap resamples to address potential overfitting, resulting in bias-corrected estimates of AUC and calibration. The model's generalizability was then confirmed through external validation on an independent dataset, with consistent performance metrics across both datasets.

Decision curve analysis (DCA) evaluates a model's clinical utility by quantifying its net benefit across probability thresholds, integrating both true positives and the cost of false positives. Unlike traditional metrics (e.g., AUC-ROC), DCA directly assesses a model's decision-making value in clinical settings.

### 2.7 Statistical analysis

Continuous variables with skewed distributions were reported as medians and interquartile ranges (IQR: P25–P75), while normally distributed variables were expressed as mean ± standard deviation (SD). Categorical variables were summarized as counts and percentages. Comparisons between survivors and non-survivors were performed using the Wilcoxon rank-sum test for non-normally distributed continuous variables and the chi-square test for categorical variables. All statistical tests were two-sided, with statistical significance defined as *p* < 0.05. Data analysis was conducted using Stata version 18.0 and R 4.3.1 software.

## 3 Results

### 3.1 Study population and cohort characteristics

Between January 2018 and March 2024, a total of 1,546 very low birth weight (VLBW) infants were admitted to the neonatal intensive care unit (NICU). After applying strict exclusion criteria (incomplete data, *n* = 4; withdrawal of care within the first 7 days of sepsis treatment, *n* = 3), a final cohort of 273 infants was enrolled. These infants were prospectively divided into a training set (*n* = 202, admitted between January 2018 and December 2022) and a validation set (*n* = 71, admitted between January 2023 and March 2024). Table 1 summarizes the baseline characteristics of both the training (primary) and test (validation) cohorts. The overall mortality rate was comparable between the two groups (*p* = 0.512). Sepsis-related mortality was observed in 20.3% in the primary cohort and 23.9% in the validation cohort.

**Table 1:** Perinatal characteristics of very low birth weight (VLBW) infants with late-onset sepsis (*N* = 273).

**Variables**	**Development cohort**			**Validation cohort**		
	**Survivor (*****n*** = **161)**	**Non-survivor (*****n*** = **41)**	**P-value**	**Survivor (*****n*** = **54)**	**Non-survivor (*****n*** = **17)**	**P-value**
Median gestation (weeks)	29.6 ± 1.5	29.0 ± 1.8	0.053	30.0 ± 1.4	28.8 ± 1.8	0.009
Median birth weight (g)	1,080 ± 136	1,068 ± 155	0.616	1,040 ± 107	986 ± 165	0.143
Gender (male/female)	(103/58)	(24/17)	0.52	(34/20)	(9/8)	0.461
Birth mode (CS/vaginal)	(80/81)	(10/31)	0.004	(29/25)	(5/12)	0.219
Median Apgar score at 1 min	8 (7~9)	8 (6~9)	0.25	8 (8~9)	8 (6~9)	0.458
Median Apgar score at 5 min	9 (8~10)	9 (8~9)	0.315	9 (8~10)	9 (8~9)	0.686
**Species of pathogens of blood culture**						
1. Gram-positive bacteria	52 (32.3%)	10 (24.4%)	0.327	13 (24.1%)	2 (11.8%)	0.278^*^
2. Gram-negative bacteria	45 (28.0%)	15 (36.6%)	0.28	18 (33.3%)	5 (29.4%)	0.7630
3. Fungemia	14 (8.7%)	3 (7.3 %)	0.777	2 (3.7%)	2 (11.8%)	0.209^*^
4. Culture negnative	50 (31.1%)	13 (31.7%)	0.936	21 (38.9%)	8 (47.1%)	0.556
Antenatal fever	6 (3.7%)	2 (4.9%)	0.508^*^	0	1 (5.9%)	0.239^*^
Antenatal steroids (%)	103 (64.0%)	26 (63.4%)	0.947	32 (59.3%)	10 (58.8%)	0.9750
Prolonged rupture of membrane (%)	67 (41.6%)	15 (36.6%)	0.558	21 (38.9%)	6 (35.3%)	0.7900
Pregnancy-induced hypertension (%)	40 (24.8%)	6 (14.3%)	0.164	11 (20.3%)	3 (17.6%)	0.556^*^
Gestational diabetes (%)	21 (13.0%)	7 (17.1%)	0.505	10 (18.5%)	5 (29.4%)	0.261^*^
Histologic chorioamnionitis (%)	3 (1.9%)	0	0.504^*^	1 (1.9%)	0	0.761^*^

^*^Fisher's exact test.

#### 3.1.1 Baseline demographic and clinical features

The study population exhibited similar baseline characteristics across both cohorts. Median gestational age was 29.4 weeks (interquartile range IQR, 28.4–30.6 weeks) in the training cohort and 29.7 weeks (IQR, 28.7–30.9 weeks) in the validation cohort (*p* = 0.917). Similarly, birth weight was comparable, with a median of 1,078 g (IQR, 1,000–1,150 g) in the training cohort and 1,024 g (IQR, 980–1,130 g) in the validation cohort (*p* = 0.116). Maternal clinical features, including gestational hypertension, gestational diabetes, prolonged rupture of membranes, antenatal fever, and antenatal steroid administration, were similarly distributed between the two groups. Neonatal characteristics, such as gender distribution, Apgar scores at 1 and 5 min, and the incidence of positive blood cultures, were also consistent across both cohorts.

##### 3.1.1.1 Gestational age and birth weight

Survivors in the training cohort had a median gestational age of 29.6 weeks (±1.5) compared to 29.0 weeks (±1.8) in non-survivors (*P* = 0.053). In the validation cohort, survivors had a significantly higher median gestational age of 30.0 weeks (±1.4) compared to 28.8 weeks (±1.8) in non-survivors (*P* = 0.009). Birth weight was slightly higher in survivors (training: 1,080 g ± 136; validation: 1,040 g ± 107) than in non-survivors (training: 1,068 g ± 155; validation: 986 g ± 165), though differences were not statistically significant (*P* = 0.616 and *P* = 0.143, respectively).

##### 3.1.1.2 Mode of delivery

Cesarean delivery was more common among survivors in the training cohort (80%) than among non-survivors (10%; *P* = 0.004). However, this association was not statistically significant in the validation cohort (survivors: 29%; non-survivors: 5%; *P* = 0.219).

##### 3.1.1.3 Pathogen distribution

In the training cohort, survivors had a higher proportion of Gram-positive pathogens (32.3%) compared to non-survivors (24.4%), while non-survivors exhibited a higher incidence of Gram-negative bacteria (36.6%) and culture-negative cases (31.7%; *P* = 0.28 for Gram-negative). In the validation cohort, survivors had a lower proportion of Gram-positive pathogens (24.1%) and a higher incidence of culture-negative cases (38.9%) compared to non-survivors (Gram-positive: 11.8%; culture-negative: 47.1%; *P* = 0.556 for culture-negative).

##### 3.1.1.4 Obstetric complications

Gestational hypertension was more common among survivors in the training cohort (24.8%) than non-survivors (14.3%; *P* = 0.164), while gestational diabetes showed no significant difference (*P* = 0.505). In the validation cohort, gestational hypertension was observed in 20.3% of survivors vs. 17.6% of non-survivors (*P* = 0.556), while gestational diabetes was more common among non-survivors (29.4%) compared to survivors (18.5%; *P* = 0.261).

### 3.2 Feature selection and dimensionality reduction

Clinical characteristics and laboratory results were assessed as potential predictors of sepsis-related mortality and included in LASSO regression analyses across different time points (Supplementary Figure S1). Distinct prognostic features were identified at each time point based on the optimal lambda values (17): (1) at 0-h time point model: nSOFA score and oxygenation index (OI) with lambda = 26.67; (2) at 6-h time point model: nSOFA score, OI, pH, lactate, and pulmonary hemorrhage with lambda = 18.39; (3) at 12-h time point model: nSOFA score, OI, and lactate with lambda = 25.82. These features were subsequently incorporated into binary logistic regression modeling, retaining only variables with a *p* < 0.05 for the final model.

**Figure 2:**
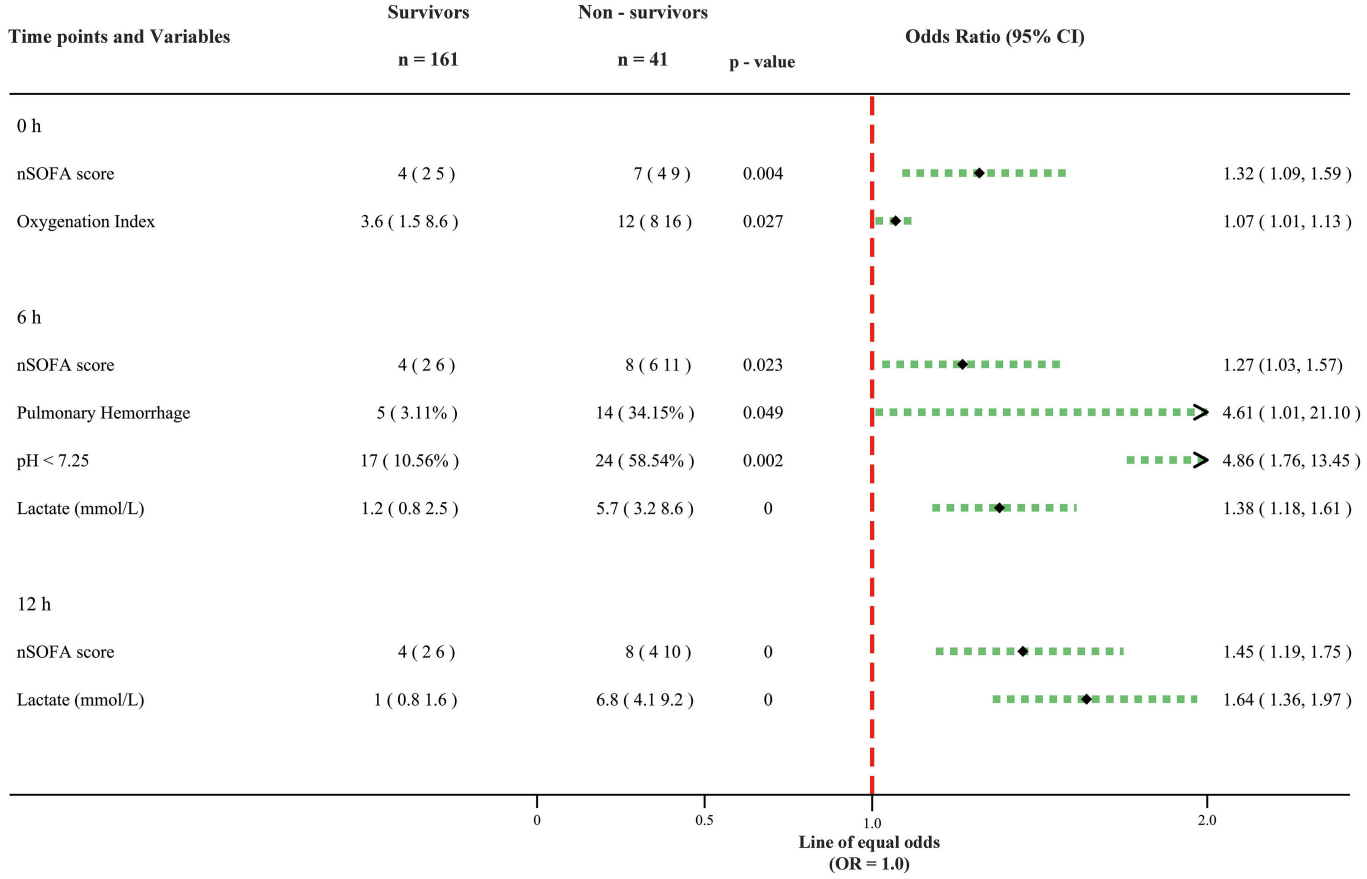
Forest plot for prognostic factors of LOS. The odd ratio of each factor is presented with a 95% confidence interval. Continuous variables are expressed as median and interquartile ranges (25%–75%). Percentages are used to express categorical variables.

At the 0-h time point, two predictors associated with mortality were identified: nSOFA score (OR: 1.32, 95% CI: 1.09–1.59) and oxygenation index (OR: 1.07, 95% CI: 1.01–1.13). In the 6-h model, four predictors emerged: nSOFA score (OR: 1.27, 95% CI: 1.03–1.57), pulmonary hemorrhage (OR: 4.61, 95% CI: 1.01–21.10), pH < 7.25 (OR: 4.86, 95% CI: 1.76–13.45), and lactate (OR: 1.38, 95% CI: 1.18–1.61). Finally, at the 12-h time point, two predictors were identified: nSOFA score (OR: 1.45, 95% CI: 1.19–1.75) and lactate (OR: 1.64, 95% CI: 1.36–1.97). Each contributory variable is represented by odds ratios (OR) and 95% confidence intervals (Figure 2).

### 3.3 Development and calibration of the predictive model

Based on the selected features, three predictive models were developed using logistic regression. The dataset was divided into a training set and a test set based on the chronological order of data collection. This temporal split was employed to mimic a real-world prospective scenario, where the model, developed using past data, is validated on more recent, unseen data.

In order to predict the risk of mortality for patients at different time points based on the logistic regression results, three different models were developed with the following variables: (1) 0 h: nSOFA score and OI; (2) 6 h: nSOFA score, pH, the lactate and pulmonary hemorrhage; (3) 12 h: nSOFA score and lactate.

The discriminative ability of the prognostic models was evaluated by calculating the area under the receiver operating characteristic (ROC) curve. Sensitivity and specificity profiles were examined using Lsens plots. Youden's index was employed to identify the optimal cutoff for distinguishing high-risk patients in the primary cohort (Supplementary Figure S2). The models demonstrated progressively enhanced predictive accuracy for mortality over time, with areas under the curve (AUCs) of 0.83 at baseline (0 h, 95% CI, 0.76–0.89), 0.92 at 6 h (95% CI, 0.87–0.97), and 0.94 at 12 h (95% CI, 0.90–0.98; Supplementary Figure S3).

**Figure 3:**
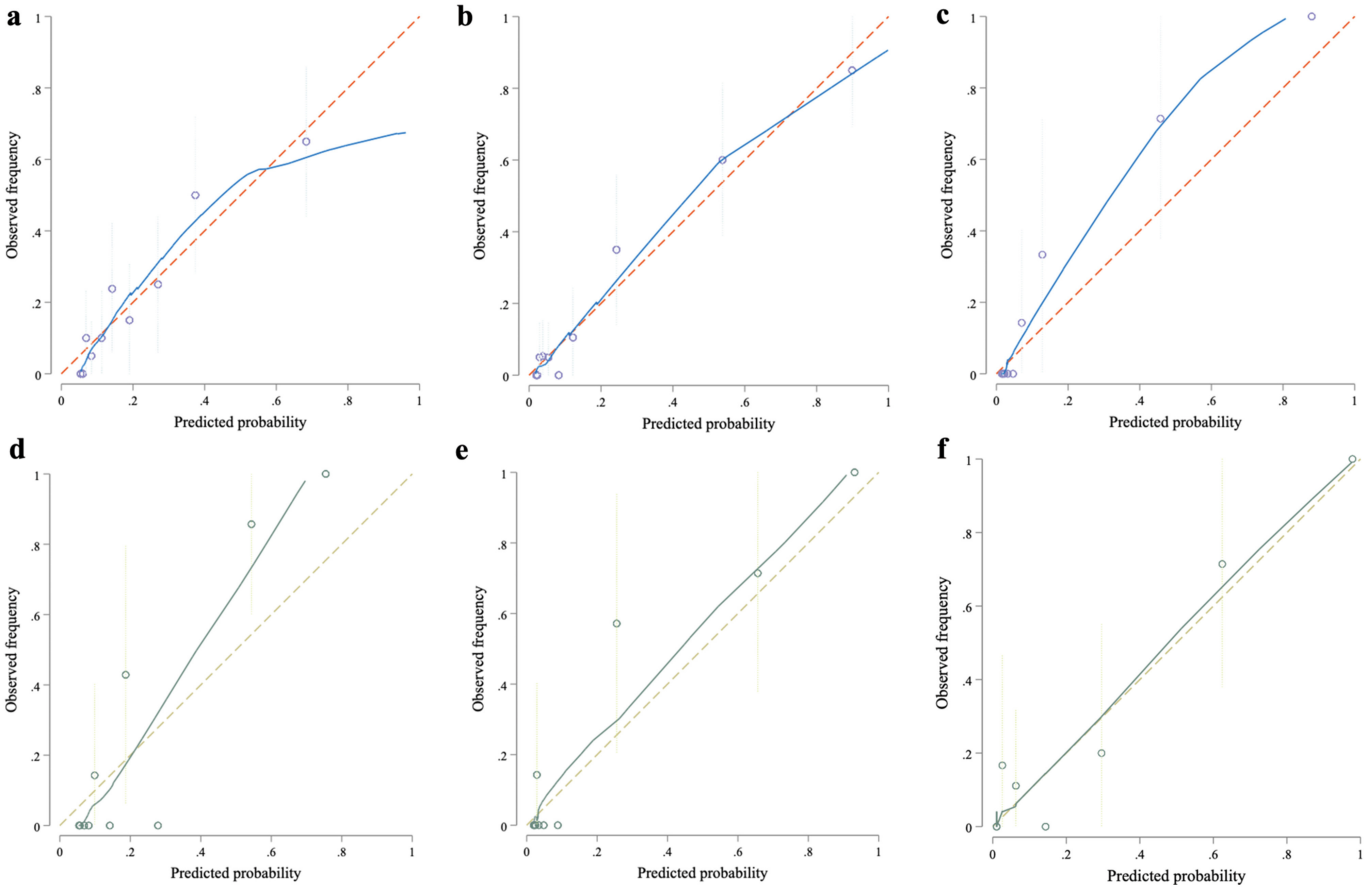
Calibration curves of the prediction models at different time points. The calibration curves evaluate the agreement between predicted and observed mortality rates at 0, 6, and 12 h within the development and validation cohorts. Each curve plots predicted probabilities against actual outcomes, with the 45-degree reference line representing ideal calibration. In both cohorts, the model demonstrates robust calibration at each time point, with predicted risks closely aligning with observed mortality rates, particularly at later time points. This consistency underscores the model's reliability and precision in mortality risk estimation across both cohorts. Panels **(a–c)** displayed calibration curves for the development cohort at 0, 6, and 12 h; while panels **(d–f)** showed the corresponding curves for the validation cohort.

The Hosmer–Lemeshow test was applied to measure the model's goodness of fit. We observed excellent agreement between expected and actual results based on the calibration plots with slopes of 1.0 (Figure 3), and no significant over- or under-prediction was detected during the Hosmer-Lemeshow test (0 h: *p* = 0.78, 6 h: *p* = 0.86; and 12 h: *p* = 0.38).

### 3.4 Nomogram construction and practical application

**Figure 4:**
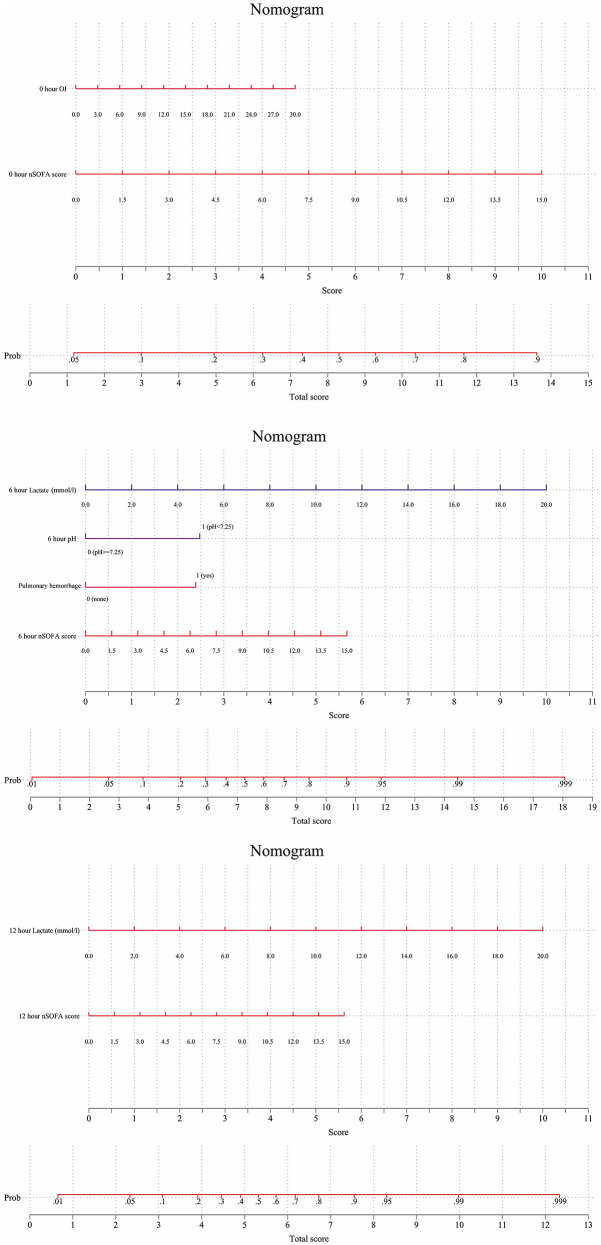
Nomogram for predicting mortality risk based on clinical variables. The nomogram indicates the mortality risk for individuals at the early stages of LOS (0, 6, and 12 h). For clinical use, the score of a variable is determined by drawing a line straight to the point axis to establish the different values. The scores of each variable are added, and the total score is located on the total score points axis. A line is drawn straight to the risk of the Prob axis to obtain the probability.

Finally, three nomograms of different times to predict the probability of mortality for individuals were developed based on the significant indicators extracted from logistic regression (Figure 4). A weighted score was assigned to each component of the nomogram; the total score was then summed to predict the risk of death for each infant. Generally, the higher the total score, the higher the mortality risk. For example, if we aim to assess the prognosis for one infant using the 6 h model, he had a level of blood lactate concentration of 6 mmol/L (3 points), pH of 7.1 (2.5 points), pulmonary hemorrhage occurred 3 h after the onset of symptoms of infection, and the nSOFA score was 12 points (4.5 points), respectively. The total score is approximately 12.5, indicating estimated mortality of 95% for this case.

### 3.5 Model performance: internal and external validation

#### 3.5.1 Internal validation

To assess the internal validity of the predictive models, we utilized bootstrap resampling with 1,000 iterations. This approach provided robust estimates, with AUC values demonstrating excellent discriminatory ability at different time points: 0.77 (95% CI: 0.76–0.89) at 0 h, 0.87 (95% CI: 0.84–0.92) at 6 h, and 0.90 (95% CI: 0.86–0.94) at 12 h post-sepsis onset in very low birth weight (VLBW) infants. Calibration plots also confirmed the strong agreement between predicted and observed mortality outcomes, with Hosmer-Lemeshow test results showing non-significant *p*-values (0 h: *p* = 0.78, 6 h: *p* = 0.86, 12 h: *p* = 0.38), indicating good model calibration (18) (Supplementary Figure S4).

#### 3.5.2 External validation

External validation was conducted in an independent cohort, where maternal COVID-19 infection was significantly more prevalent (89%) compared to the training cohort (0.01%). Despite these differences, the models exhibited superior discriminatory ability with AUC values of 0.95 (95% CI: 0.89–1.00) at 0 h, 0.95 (95% CI: 0.88–1.00) at 6 h, and 0.97 (95% CI: 0.94–1.00) at 12 h. Hosmer-Lemeshow test results also remained non-significant (0 h: *p* = 0.42, 6 h: *p* = 0.22, 12 h: *p* = 0.50), confirming good model calibration in this independent cohort (Figure 3).

#### 3.5.3 Decision curve analysis (DCA) for clinical utility of sepsis mortality prediction models

**Figure 5:**
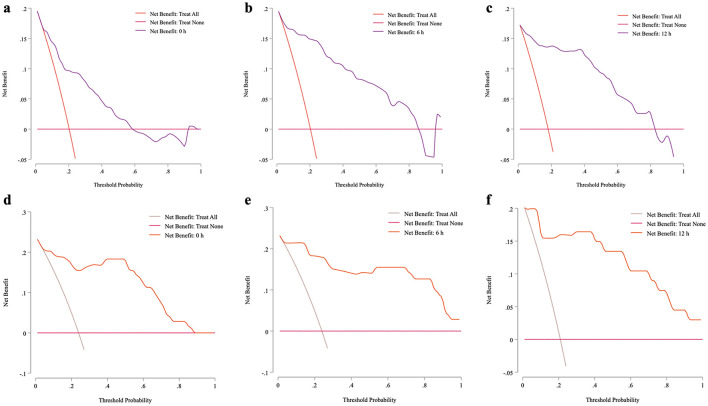
Decision curve analysis (DCA) for the prediction models in Development cohort and Validation cohort at different time points. The decision curve analysis (DCA) plots illustrate the net benefit of the mortality prediction model at 0, 6, and 12 h across both development and validation cohorts, evaluated over a range of threshold probabilities. Net benefit, represented on the *y*-axis, reflects the balance between true positive identifications and the avoidance of false positives, with the *x*-axis representing threshold probability. At each time point, the model demonstrates a greater net benefit than alternative strategies (such as treating all or no patients), underscoring its clinical utility in identifying high-risk patients. Notably, net benefit increases at later time points, suggesting enhanced decision support as prediction time progresses. Panels **(a–c)** depicted DCA curves for the development cohort at 0, 6, and 12 h, respectively; while panels **(d–f)** illustrated the corresponding curves for the validation cohort.

To evaluate the clinical utility of timepoint-specific prediction models (0 h, 6 h, 12 h) for sepsis mortality in VLBW infants, we performed decision curve analysis (DCA) across a range of risk thresholds (Figure 5). The 12% risk threshold emerged as optimal for all models, balancing harm-to-benefit trade-offs (3:22 ratio; i.e., avoiding 3 deaths per 22 unnecessary interventions) and maximizing net clinical benefit (Supplementary Figure 5).

##### 3.5.3.1 Zero-hour model

At the 12% threshold, the model classified 49.01% (95% CI: 30.7–100) of patients as high risk, achieving a net benefit of 0.47 (95% CI: 0.25–0.61), with 47% of deaths correctly identified (sensitivity) and 67% of non-fatal cases accurately excluded (specificity).

##### 3.5.3.2 Six-hour model

Refined risk stratification reduced high-risk classifications to 28.7% (95% CI: 20.8–38.6) while maintaining a net benefit of 0.47 (95% CI: 0.28–0.62). Specificity improved to 81% (95% CI: 0.68–0.88) without compromising sensitivity (47%, 95% CI: 0.28–0.62).

##### 3.5.3.3 Twelve-hour model

Precision further improved, narrowing high-risk classifications to 22.7% (95% CI: 14.95–34.02) with specificity of 80.3% (95% CI: 0.648–0.907). While net benefit remained clinically meaningful (0.38, 95% CI: 0.03–0.57), sensitivity variability increased (38.1%, 95% CI: 0.034–0.574), reflecting heterogeneity in late-stage sepsis trajectories.

The consistent 12% threshold across all models underscores its reliability for early intervention decisions. These nomograms provide actionable guidance for initiating or intensifying interventions in VLBW infants with late-onset sepsis. By identifying 38–47% of fatal outcomes and avoiding over-treatment in 67–81% of non-fatal cases, the models align with harm-to-benefit preferences in neonatal critical care.

#### 3.5.4 Interpretation of superior discrimination in the testing cohort

The striking disparity in maternal COVID-19 infection rates between the testing and training cohorts may provide a compelling explanation for the superior discrimination observed in the testing cohort. Notably, 89% of mothers in the testing cohort had COVID-19 during pregnancy, compared to only 1% in the training cohort. This stark difference likely influenced neonatal outcomes, particularly in preterm infants. Maternal COVID-19 infection is known to profoundly affect fetal and neonatal physiology, potentially altering the trajectory of conditions such as LOS in preterm neonates. These maternal-infection-induced physiological changes could have contributed to the enhanced discriminatory power seen in the testing cohort.

## 4 Discussion

The primary goal of this study was to develop and validate a nomogram-based scoring system for predicting mortality in VLBW infants with LOS, a cohort known for its high morbidity and mortality rates. In this discussion, we reflect on the importance of our findings, emphasizing the novel aspects of the study, the unexpected performance in the test cohort, and how these results extend the current knowledge of sepsis in VLBW infants. Additionally, we will evaluate the clinical utility of the model, its implications for practice, and potential avenues for future research.

### 4.1 Innovation and clinical importance

A notable innovation of this study is the dynamic, time-dependent approach used to predict mortality risk in neonates with LOS. This is particularly significant given that existing models tend to rely on static parameters, often limited to a single time point. Our model, by incorporating three distinct time points (0 h, 6 h, 12 h) after the onset of sepsis, addresses a critical gap in neonatal care, where the clinical status of VLBW infants can rapidly deteriorate.

Moreover, this approach aligns with recent trends toward dynamic risk assessment in critically ill patients, recognizing the importance of continuous monitoring and time-sensitive interventions. In neonatal intensive care units (NICUs), where clinical deterioration can occur rapidly in VLBW infants with sepsis, having a tool that adapts to changes in physiological parameters over time allows for more timely interventions. This dynamic approach aligns with the latest advancements in neonatal sepsis management, which emphasize the need for continuous monitoring and personalized interventions. The model's ability to adapt to changes in the neonate's condition over time could lead to improved survival outcomes by allowing clinicians to initiate treatment earlier in the disease course when signs of deterioration first become apparent (6).

### 4.2 Model performance and unexpected findings

One of the most surprising and intriguing findings of this study was the model's superior performance in the external validation cohort. At all three time points, the area under the curve (AUC) values were significantly higher than those in the development cohort. In the test cohort, the AUC values were 0.95 at 0 h, 0.95 at 6 h, and 0.97 at 12 h, demonstrating the model's exceptional ability to distinguish between survivors and non-survivors.

Upon further analysis, we identified a significant difference in the clinical characteristics between the development and test cohorts—specifically, the prevalence of maternal COVID-19 infection. In the test cohort, 89% of mothers had a documented COVID-19 infection during pregnancy, while only 1% of mothers in the development cohort had the same. To evaluate whether maternal COVID-19 infection influenced the predictive performance of our model, we included it as a binary variable (infected vs. non-infected) in multivariable logistic regression models at three distinct time points: 0 h, 6 h, and 12 h post-sepsis onset. The adjusted odds ratios (ORs) and corresponding 95% confidence intervals (CIs) were as follows: (1) 0 h model: OR = 1.20, *p* = 0.696 (95% CI: 0.485, 2.956); (2) 6 h model: OR = 1.90, *p* = 0.228 (95% CI: 0.659, 5.727); (3) 12 h model: OR = 2.55, *p* = 0.08 (95% CI: 0.894, 7.273). The lack of statistical significance for maternal COVID-19 infection in our models may be attributable to the presence of stronger predictors of neonatal sepsis mortality. Previous studies have highlighted gestational age, birth weight, inflammatory biomarkers, and organ dysfunction as more robust determinants of neonatal outcomes compared to maternal infection alone. Given the multifactorial nature of neonatal sepsis, maternal COVID-19 infection may exert indirect effects that are attenuated when adjusting for these stronger predictors (19). Emerging research suggests that maternal COVID-19 infection can induce persistent alterations in later fetal immune programming, potentially increasing susceptibility to more severe infections such as sepsis in life (20, 21). The heightened systemic inflammation and immune dysregulation seen in infants born to mothers with COVID-19 could explain why the model performed better, particularly at the 12-h time point, where inflammatory markers played a key role in predicting mortality.

This stark contrast likely played a crucial role in the model's superior discrimination in the test cohort. This finding not only highlights the adaptive nature of the model but also underscores the importance of considering maternal factors—such as viral infections—in future predictive models. As the COVID-19 pandemic continues to affect neonatal outcomes, the necessity for predictive models that consider maternal-infant interactions is becoming evident (22). Future studies should explore whether maternal COVID-19 infection severity, viral load, and timing of infection during pregnancy (early, mid, or late trimester) differentially impact neonatal sepsis risk and other neonatal outcomes.

### 4.3 Future directions and implications

The implications of these findings are 2fold. First, the study highlights the importance of dynamic modeling for predicting sepsis. It suggests that future models should include time-dependent variables to accurately represent the progressive nature of sepsis in critically ill neonates. Second, these findings raise important questions about how maternal infections, especially COVID-19, influence neonatal outcomes (20). Further research is needed to explore the connections between maternal viral infections and the susceptibility of neonates to sepsis, particularly how these infections might change the neonatal immune response during critical periods, such as late-onset sepsis (23).

Additionally, the model's performence in the test cohort, which had a high rate of maternal COVID-19 infections, suggests that future predictive models should take maternal health factors into account as potential influences on neonatal outcomes. This finding could have significant clinical implications, not only for managing sepsis in VLBW infants but also for our approach to perinatal care regarding maternal infections.

### 4.4 Limitations

This study has several limitations that need to be recognized. Firstly, being a retrospective analysis, it is susceptible to inherent biases like selection bias and information bias, which could compromise the accuracy and generalizability of the results. Secondly, since data were gathered from a single center, this may restrict the external validity and complicate the ability to apply the findings to various healthcare settings. Thirdly, although we considered major confounders, there may still be unmeasured variables, such as genetic factors or differences in clinical practices, that could have impacted the outcomes. Finally, while the effects of maternal COVID-19 infection on neonatal outcomes are significant, further investigation through prospective studies is necessary to confirm its role and influence on late-onset sepsis and overall neonatal health outcomes. Future research, particularly involving larger, multicenter cohorts with longitudinal follow-up, will be essential to address these limitations and validate the strength of our findings.

## 5 Conclusion

This study presents a novel, time-sensitive predictive model for mortality in VLBW infants with late-onset sepsis, validated in both internal and external cohorts. Moreover, the temporal delineation of the cohorts—where the training cohort largely represents pre-pandemic cases and the testing cohort reflects the pandemic era—further underscores this novel discovery. The unique pathophysiological landscape created by maternal COVID-19 during the pandemic may have heightened the clarity of risk stratification in neonates, allowing for more precise identification of those at higher risk for sepsis-related death. This temporal and epidemiological distinction highlights the broader impact of maternal health on neonatal outcomes and emphasizes the need for adjusted predictive models in the context of emerging infectious diseases.

## Data Availability

The datasets generated during this study are being deposited in the Figshare repository (https://figshare.com/), with a unique Digital Object Identifier (DOI) to be assigned upon article acceptance. Interim access to the data can be granted by contacting the corresponding author under a data-sharing agreement.
