# Supplementary material for: Enhanced predictive accuracy of mortality in VLBW infants with late-onset sepsis through a time-specific nomogram
Source: Front Public Health. 2025 Apr 2;13:1548695. doi: 10.3389/fpubh.2025.1548695 (PMC11999929; doi:10.3389/fpubh.2025.1548695)
Supplement: Supplementary file 1 [file Data_Sheet_1.pdf]

Supplementary Material

Table S1. Variables in the equation on LASSO regression.

Variable	Variable types	Variable assignment
**Type of respiratory support equipment**	Categorical variables	0: non;<br>1: heated humidified HFNC<br>2: non- invasive respiratory support<br>3: invasive respiratory support
**Species of pathogens of blood culture**	Categorical variables	1: Gram-negative Bacterial<br>2: Gram-positive Bacterial<br>3: Fungi
**Lethargy[1]**	Categorical variables	0: non<br>1: yes
**Perfusion of the skin with microcirculatory irregularities[1]**	Categorical variables	0: non<br>1: yes
**Tachycardia or bradycardia[2]**	Categorical variables	0: 120~180b/min<br>1: ≥180b/min<br>2: <120b/min
**Frequent episodes of apnoea[3]**	Categorical variables	0: non<br>1: yes
**Sepsis shock[4]**	Categorical variables	0: non<br>1: yes
**nSOFA score**	Continuous variables	
**Glu (mmol/l)**	Continuous variables	
**Lac (mmol/l)**	Continuous variables	

**Blood sodium (mmol/l)**	Categorical variables	0: 135~145
		1: 134~130
		2: <130
		3: >145
**Oxygenation index**	Continuous variables	
**WBC**[1]	Categorical variables	0: $20.0*10^9$ ~ $5.0*10^9$
		1: $<5.0*10^9$
		2: $\geq 20.0*10^9$
**Neutrophil absolute value**[1]	Categorical variables	0: $\geq 0.5*10^9$
		1: $< 0.5*10^9$
**PCT (ng/ml)**[1]	Categorical variables	0: < 0.5
		1: ≥ 0.5
**CRP (mg/L)**[5]	Categorical variables	0: < 10.0
		1: ≥ 10.0
**pH**	Categorical variables	0: ≥ 7.25
		1: < 7.25
**NEC**[6]	Categorical variables	0: none or I grade
		1: ≥ II A
**Pulmonary hemorrhage**[7]	Categorical variables	0: none
		1: yes

Pulmonary hemorrhage Diagnostic Criteria:

a. Clinical Presentation: Sudden onset of respiratory distress (e.g., tachypnea, grunting) and/or blood-stained secretions in tracheal aspirates or endotracheal tubes.

b. Radiographic Evidence: Chest X-rays showing patchy or diffuse alveolar infiltrates, consolidations, or pleural effusions, with findings consistent with alveolar haemorrhage.

References:

1. Jiang S, Yang C, Yang C, Yan W, Shah V, Shah PS, Lee SK, Yang Y, Cao Y: **Epidemiology and microbiology of late-onset sepsis among preterm infants in China, 2015-2018: A cohort study**. *Int J Infect Dis* 2020, **96**:1-9.
2. Goh GL, Lim CSE, Sultana R, De La Puerta R, Rajadurai VS, Yeo KT: **Risk Factors for Mortality From Late-Onset Sepsis Among Preterm Very-Low-Birthweight Infants: A Single-Center Cohort Study From Singapore**. *Frontiers in pediatrics* 2021, **9**:801955.
3. Eichenwald EC: **Apnea of Prematurity**. *Pediatrics* 2016, **137**(1).
4. Singer M, Deutschman CS, Seymour CW, Shankar-Hari M, Annane D, Bauer M, Bellomo R, Bernard GR, Chiche JD, Coopersmith CM *et al*: **The Third International Consensus Definitions for Sepsis and Septic Shock (Sepsis-3)**. *Jama* 2016, **315**(8):801-810.
5. Balayan S, Chauhan N, Chandra R, Kuchhal NK, Jain U: **Recent advances in developing biosensing based platforms for neonatal sepsis**. *Biosens Bioelectron* 2020, **169**:112552.
6. Uauy RD, Fanaroff AA, Korones SB, Phillips EA, Phillips JB, Wright LL: **Necrotizing enterocolitis in very low birth weight infants: biodemographic and clinical correlates. National Institute of Child Health and Human Development Neonatal Research Network**. *J Pediatr* 1991, **119**(4):630-638.
7. Wang TT, Zhou M, Hu XF, Liu JQ: **Perinatal risk factors for pulmonary hemorrhage in extremely low-birth-weight infants**. *World J Pediatr* 2020, **16**(3):299-304.

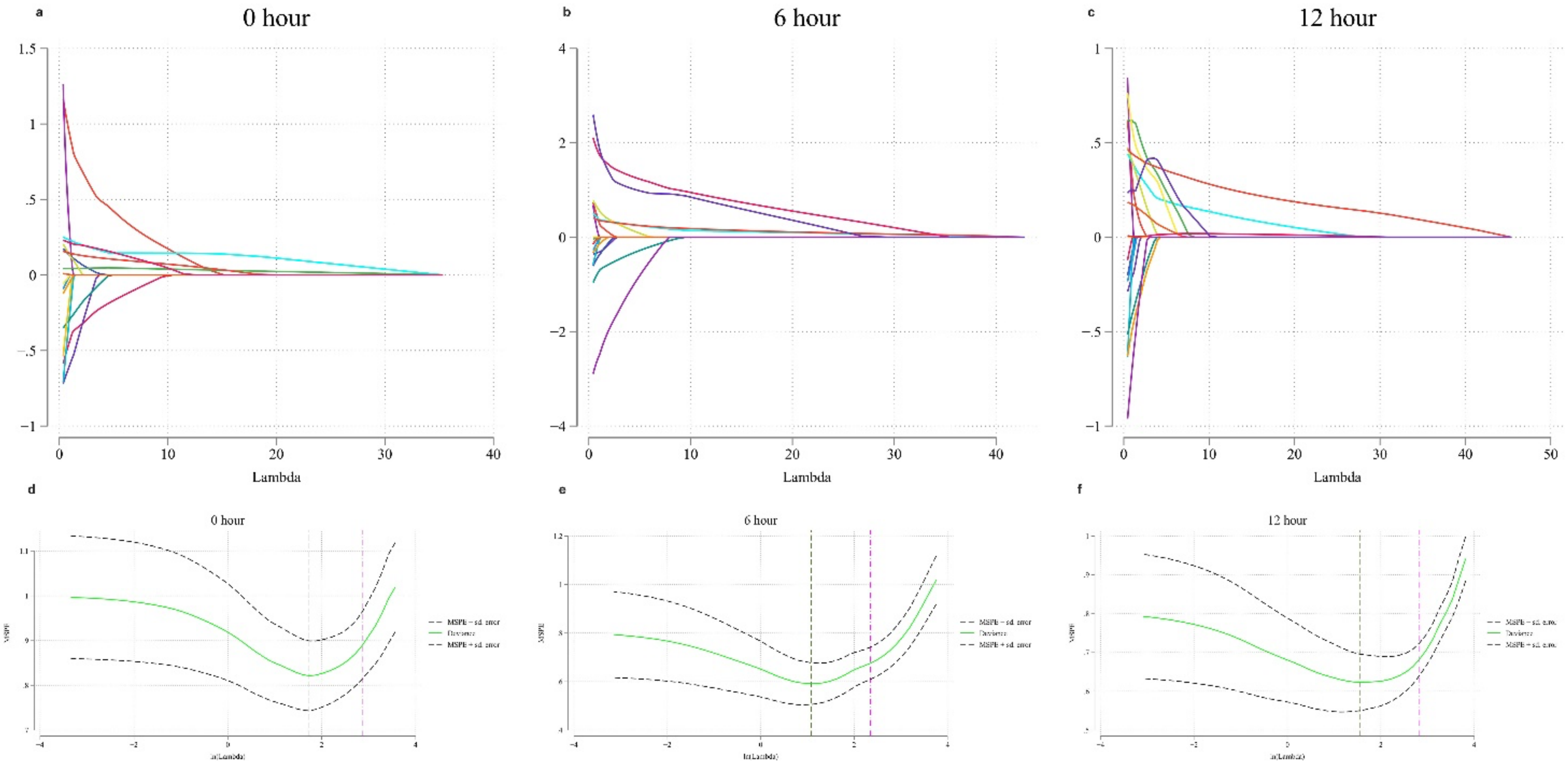

Supplemental Figure S1. Lasso regression path diagram of different timepoints.

a-c: Lambda was used to monitor and control the complexity of Lasso regression.

d-f: On the basis of lambda values established by 10-fold cross-validation, candiate factors have been chosen.

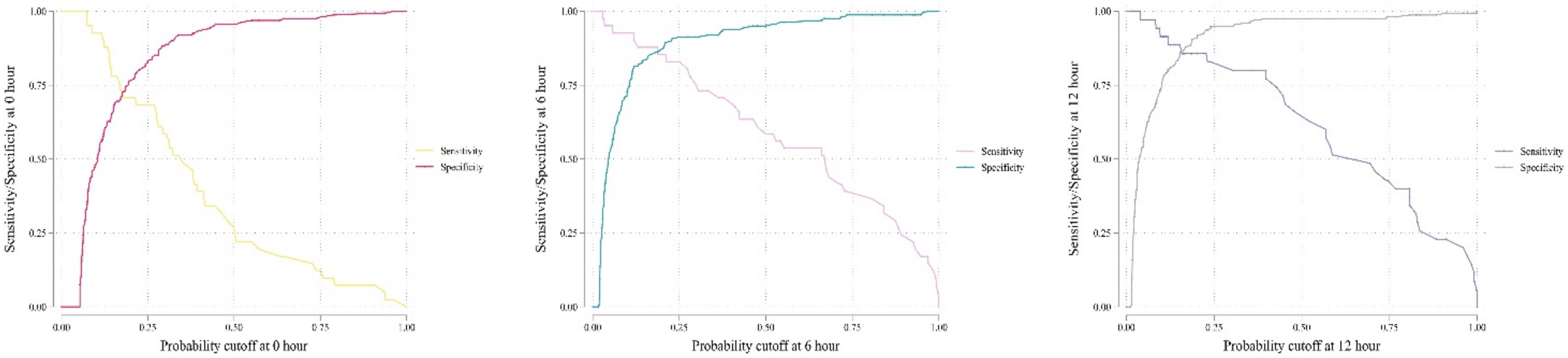

Supplemental Figure S2. The Lsens plots presented sensitivity and specificity of the prognostic models. It was determined that Youden's index provided the best cutoff value for identification of patients with a high mortality risk in the primary corhort.

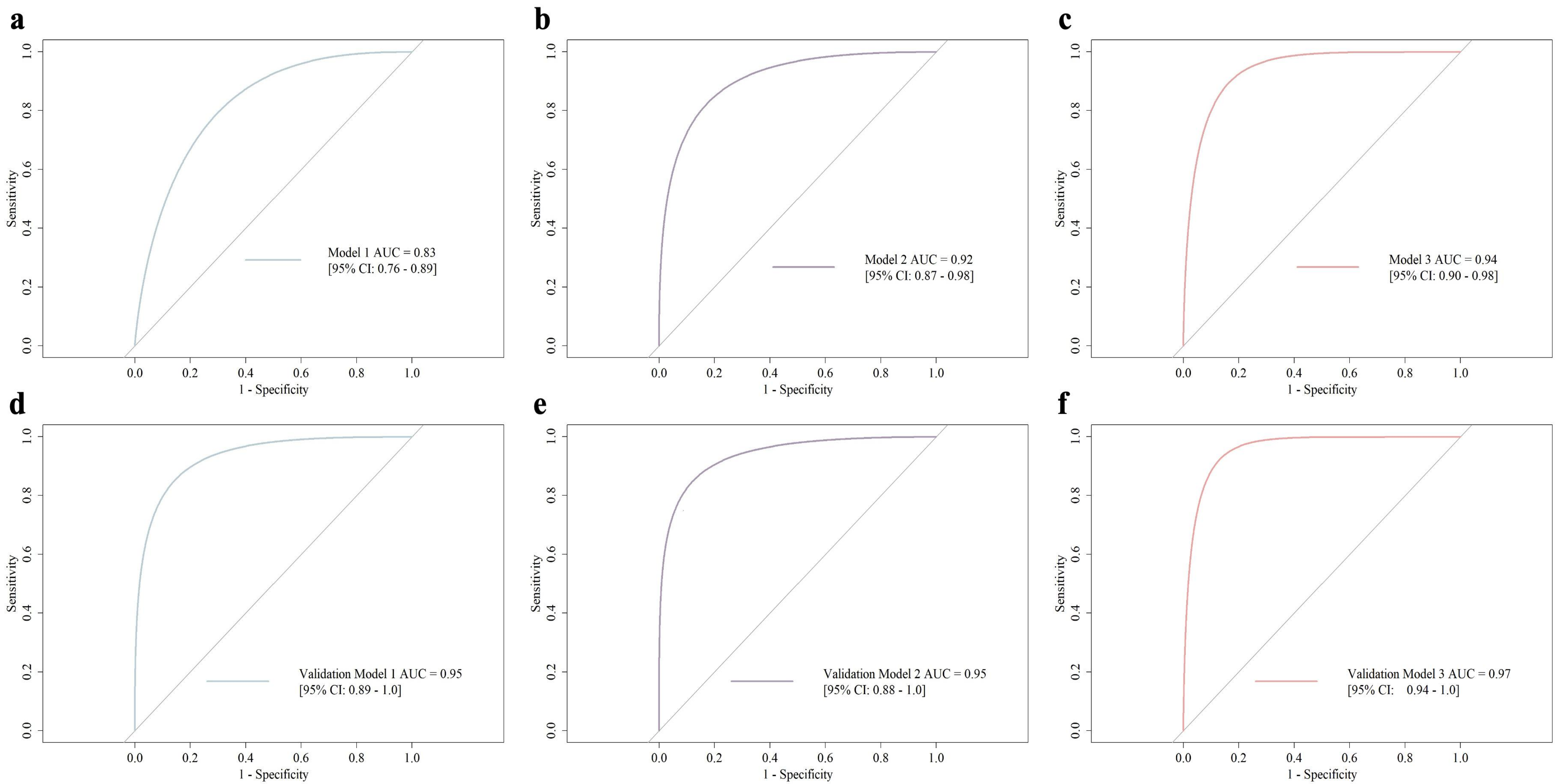

Supplemental Figure S3. Receiver Operating Characteristic (ROC) Curves for Hortality Prediction in /evelopment and Validation cohorts at different time points. The ROC curves illustrate the discriminatory performance of the mortality prediction model at three time points (0 h, 6 h, and 12 h) in both development and validation cohorts. Sensitivity and specificity were assessed across varying thresholds, demonstrating increased predict ive accuracy over time. Panels a– c shown the ROC curves for the development cohort at 0 h, 6 h, and 12 h, respectively; panels d– f represented the ROC curves for the validation cohort at the corresponding time points.

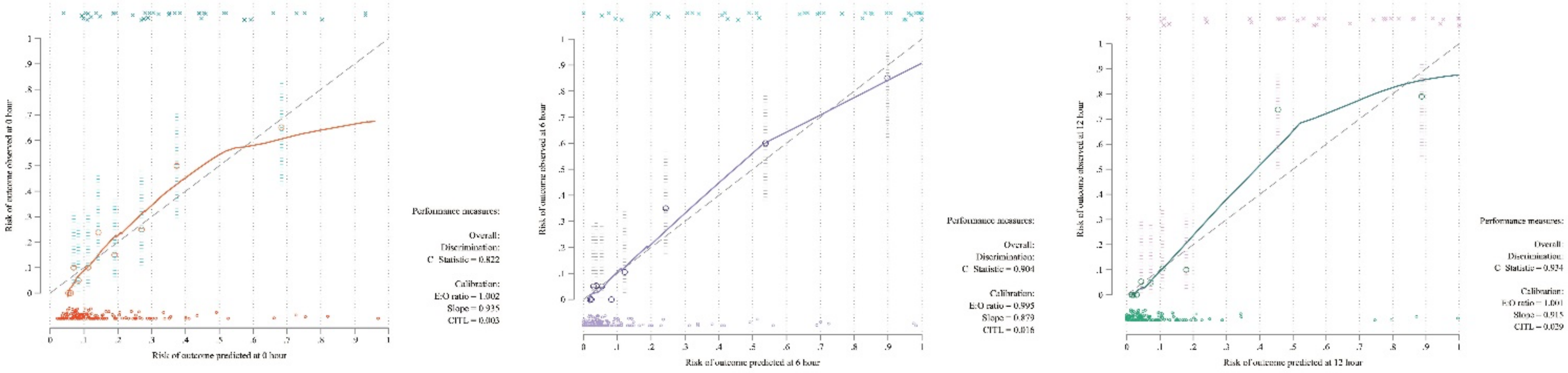

Supplemental Figure S4. The bootstrap cross-validation approach was used for internal validation of the updated prognostic models. Discrimination is represented by the C-statistic. Calibration is presented as E:O ratio, slope, and CITL.

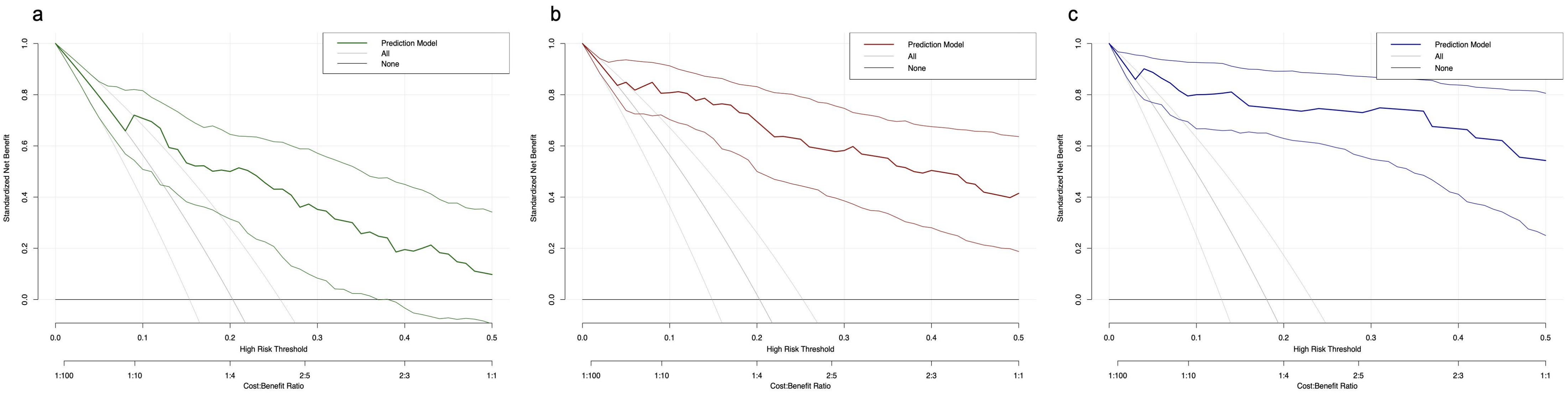

Supplemental Figure S5 Decision Curve Analysis for Predictive Models at Different Time Points
(a) 0-hour model, (b) 6-hour model, (c) 12-hour model.
Decision curve analysis (DCA) illustrates the standardized net benefit across various high-risk thresholds.
The y-axis represents the standardized net benefit, while the x-axis denotes the high-risk threshold.
Solid colored lines indicate the performance of predictive models at each time point.
Gray lines represent the net benefit of treating all patients ("All") or treating none ("None").
The corresponding cost-benefit ratio for each threshold is displayed along the x-axis.
